# External Pancreatic Juice Drainage Through a Percutaneous Endoscopic Drainage Tube for the Patient With a Postoperative Pancreatic Juice Leakage

**DOI:** 10.1155/DTE.5.137

**Published:** 1999

**Authors:** Yukio Nishiguchi, Hiroji Nishino, Kazuhiko Yoshikawa, Mikio Nakamura, Osamu Takaishi, Masatsugu Shiba, Hajime Nakamura, Tsutomu Nomura, Katsumi Ikeda, Masaichi Ohira, Takeshi Asai, Shigehito Yamagata, Kazuhiro Takeuchi, Michio Sowa, Noriko Suzuki

**Affiliations:** 1 First Department of Surgery Osaka City University Medical School 1-4-3, Asahi-machi Abeno-ku Osaka 545-8585 Japan; 2 Third Department of Internal Medicine Osaka City University Medical School 1-4-3, Asahi-machi Abeno-ku Osaka 545-8585 Japan

## Abstract

Percutaneous endoscopic gastrostomy (PEG) has been widely accepted for patients who have no swallowing ability but have an intact gut. Its clinical application is mainly for nutritional support and decompression of the intestine in patients with bowel obstruction. In this paper, we report external pancreatic juice drainage through a percutaneous endoscopic drainage tube in a patient with postoperative pancreatic juice leakage. Soon after this procedure, pancreatic juice leakage subsided. This procedure was minimally invasive for the patient and may be a new application of PEG to maintain the good quality of life (QOL) in a patient with pancreatic juice leakage.


*Diagnostic and Therapeutic Endoscopy*, Vol. 5, pp. 137–140
Reprints available directly from the publisher
Photocopying permitted by license only

© 1999 OPA (Overseas Publishers Association) N.V.
Published by license under
the Harwood Academic Publishers imprint,
part of The Gordon and Breach Publishing Group.
Printed in Singapore.

### External Pancreatic Juice Drainage Through a Percutaneous Endoscopic Drainage Tube for the Patient with a Postoperative Pancreatic Juice Leakage

YUKIO NISHIGUCHI[a,*], HIROJI NISHINO[a], KAZUHIKO YOSHIKAWA[a], MIKIO NAKAMURA[b], OSAMU TAKAISHI[b], MASATSUGU SHIBA[b], HAJIME NAKAMURA[b], TSUTOMU NOMURA[b], KATSUMI IKEDA[a], MASAICHI OHIRA[a], TAKESHI ASAI[a], SHIGEHITO YAMAGATA[a], KAZUHIRO TAKEUCHI[a], MICHIO SOWA[a] and NORIKO SUZUKI[b]

[a]*First Department of Surgery,* [b]*Third Department of Internal Medicine, Osaka City University Medical School, 1-4-3, Asahi-machi, Abeno-ku, Osaka-City, Osaka 545-8585, Japan*

*(Received 12 June 1998; In final form 4 August 1998)*

**Percutaneous endoscopic gastrostomy (PEG) has been widely accepted for patients who have no swallowing ability but have an intact gut. Its clinical application is mainly for nutritional support and decompression of the intestine in patients with bowel obstruction. In this paper, we report external pancreatic juice drainage through a percutaneous endoscopic drainage tube in a patient with postoperative pancreatic juice leakage. Soon after this procedure, pancreatic juice leakage subsided. This procedure was minimally invasive for the patient and may be a new application of PEG to maintain the good quality of life (QOL) in a patient with pancreatic juice leakage.**

*Keywords:* Pancreas juice leakage, ERPD, ENPD, PEG

### INTRODUCTION

Pancreatic juice leakage is a most troublesome complication after pancreatic surgery. External drainage of the pancreas juice is a common choice of this treatment, but this leakage is not always successfully treated conservatively. Subsequent surgical treatment such as distal pancreatectomy or fistulo-enterostomy is commonly chosen in many cases.

There are no reports describing pancreatic juice suction via an endoscopic naso-pancreatico drainage (ENPD) tube in a patient with pancreatic juice leakage. In this report, we describe successful external pancreatic juice drainage through a percutaneous endoscopic drainage tube in a patient with a postoperative pancreatic juice leakage.

### CASE REPORT

A 52-year-old man presented with a persistent post-prandial back pain. Ultrasonography, computed

* Corresponding author. Tel.: (06) 645 3838. Fax: (06) 646 6450.

tomography and endoscopic retrograde cholangiopancreatography (ERCP) revealed a tumor in the pancreatic head. Preoperatively, obstructive jaundice occurred and percutaneous transhepatic gallbladder drainage (PTGBD) was performed. At laparotomy, fine needle aspiration biopsy indicated that the tumor was not malignant. Cholecystectomy and choledocho-duodenal anastomosis were performed. Fourteen days postoperatively, pancreatic juice began to be drained and continued to be drained from the surgical drainage about 140 ml/day, for about one week.

ERCP revealed almost total obstruction of the main pancreatic duct at the pancreatic head. Endoscopic retrograde pancreatico drainage (ERPD) was performed as previously described. Then an ENPD tube was inserted through the ERPD tube (Fig. 1), and the end of the ENPD tube placed in the lumen of the stomach (Fig. 2). A 20 French PEG tube was placed using a previously described technique [1,2].Then the tip of the ENPD tube was introduced into the gastrostomy tube from the gastric lumen to the internal bumper of the gastrostomy tube. Therefore, the ENPD tube was correctly placed through the PEG tube (Fig. 3). After placement of the tubes, an average of 500 ml of pancreatic juice was obtained through the ENPD tube daily. Soon after placement of the tubes, pancreatic juice leakage from the surgical drainage disappeared.

Forty-eight days postoperatively, contact media from the ENPD tube revealed that there was no further leakage from the main pancreatic duct, then the ENPD tube and gastrostomy tube were

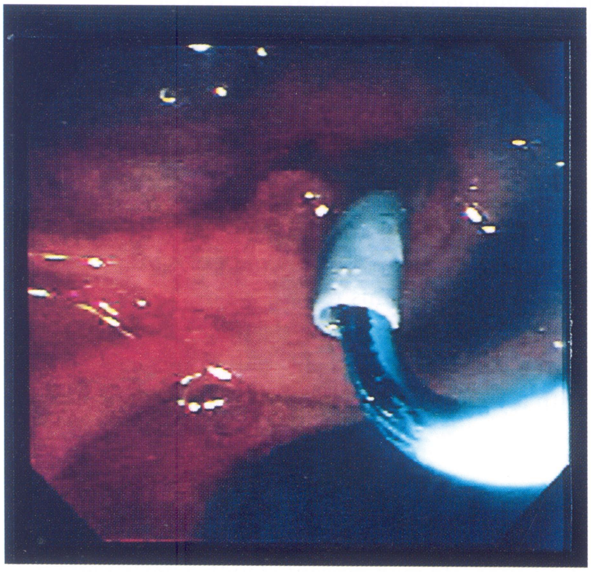

FIGURE 1 Endoscopic photograph of ENPD tube through ERPD tube. ENPD tube was inserted in ERPD tube (pancreatic duct stenting tube) through the stenotic portion of the main pancreatic duct.

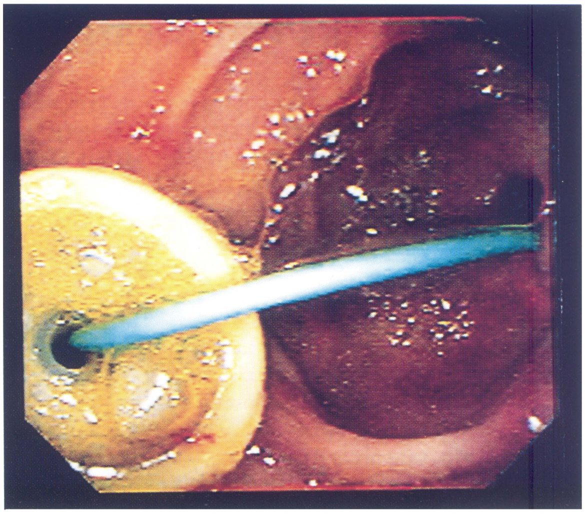

FIGURE 2 Endoscopic photograph of ENPD tube through the internal bumper of PEG tube. The ENPD tube was introduced into the gastrostomy tube from the gastric lumen to the internal bumper of the gastrostomy tube.

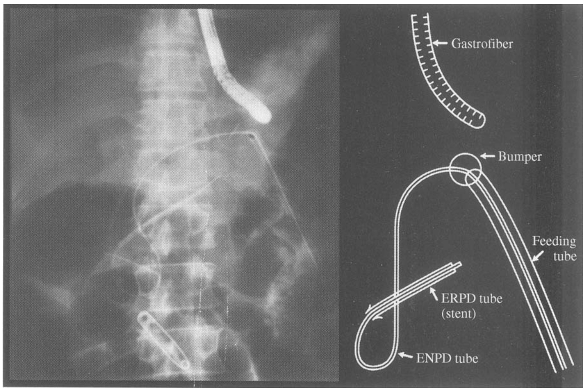

FIGURE 3 Radiogram of the placed tubes. The ENPD tube in the ERPD tube was correctly placed through the PEG (feeding) tube.

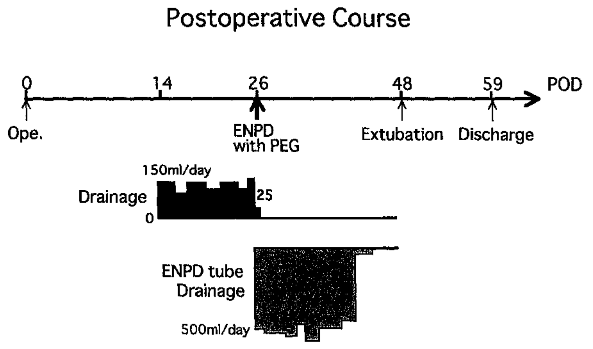

FIGURE 4 Postoperative course. After placement of the tubes, an average of 500 ml of pancreatic juice was obtained through the ENPD tube daily. Soon after placement of the tubes, pancreatic juice leakage from the surgical drainage disappeared.

removed. Fifty-six days postoperatively, the patient was discharged and he is now doing well and working in good health (Fig. 4).

### DISCUSSION

Pancreatic juice leakage is a most troublesome complication of pancreas surgery [3]. As conservative treatment of pancreatic juice leakage, protease inhibitor with fasting and total parenteral nutrition (TPN) are commonly chosen [4]. Somatostatin analog has recently been used for this treatment and good results have been obtained [5]. However, when the amount of pancreatic juice leakage continues over 200 ml/day and persists over three months, surgical treatment is chosen. Pancreaticojejunal anastomosis or partial resection of the pancreas is usually chosen, but there are sometimes troublesome procedures.

Pancreatic cyst is a common complication causing late pancreatic juice leakage [6,7]. For such pancreatic cyst, cyst-enterostomy or distal pancreatectomy is usually chosen. Recently, endoscopic cyst-gastric fistula formation has been reported as a less invasive treatment and has been successfully performed [8–10]. In any case, it is important in ERCP to determine whether the main pancreatic duct has become stenotic.

It is reasonable to perform stenting of the stenotic portion of the pancreatic duct for the treatment of pancreatic juice leakage with the pancreatic duct stenosis [11,12]. Furthermore, suction of the pancreatic juice from the distal portion of the pancreatic duct is more effective for the treatment of these patients. In our case, surgical pancreatic biopsy made obstruction of the stenotic portion of the pancreatic duct because of the peripancreatic duct inflammation. An ERPD tube was inserted to the obstructed stenotic portion of the pancreatic duct, and then, an ERPD tube was inserted through the ERPD tube. Furthermore, this patient was the special patient who did not tolerate the insertion of the tube through the nose, therefore, an ENPD tube was drained via the PEG tube and suctioned. Finally, this patient maintained a good quality of life and pancreatic juice leakage subsided soon after this treatment.

In conclusion, our case demonstrates the usefulness and good quality of life during external pancreatic juice drainage through a percutaneous endoscopic drainage tube in a patient with postoperative pancreatic juice leakage. This new application of PEG is recommended for pancreatic juice leakage.

### *References*

[1] Gauderer, M.W.L. and Ponsky, J.L.P. Percutaneous endoscopic gastrostomy: Indication, limitations, techniques, and results. *World J. Surg.* 1989; **13**: 165–170.
[2] Nishiguchi, Y., Fuyuhiro, Y., Lee, J.T. *et al.* Percutaneous endoscopic gastrostomy, duodenostomy and jejunostomy. *Diag. Therapeutic Endosc.* 1994; **1**: 37–43.
[3] Wolfsen, H.C., Kozarek, R.A., Ball, T.J. *et al.* Pancreaticoenteric fistula. *J. Clin. Gastroenterol.* 1992; **14**: 117–121.
[4] Uchiyama, T., Yamamoto, T., Mizuta, E., *et al.* Pancreatic ascites - a collected review of 37 cases in Japan. *Hepato-Gasatroenterol.* 1989; **36**: 244–249.
[5] Lansden, F.T., Adams, D.B. and Anderson, M.C. Treatment of external pancreatic fistulas with somatostatin. *Am. Surg.* 1989; **55**: 695–698.
[6] Adams, D.B. and Anderson, M.C. Changing concepts in the surgical management of pancreatic pseudocysts. *Am. Surg.* 1991; **58**: 173–180.
[7] Wilson, C. Management of the later complications of severe acute pancreatitis -pseudocyst, abscess and fistula. *Eur. J. Gastroenterology & Hepatology* 1997; **9**: 117–121.
[8] Ho, C.S. and Taylor, B. Percutaneous transgastric drainage for pancreatic pseudocyst. *AJR* 1984; **143**: 623–625.

[9] Kuligowska, E. and Olsen, W.L. Pancreatic pseudocysts drained through a percutaneous transgastric approach. *Radiology* 1985; **154**: 79–82.

[10] Deviere, J., Bueso, H., Baize, M. *et al.* Complete disruption of the main pancreatic duct: endoscopic management. *Gastrointestinal Endoscopy* 1995; **42**: 445–451.

[11] Kozarek, R.A., Ball, T.J., Patterson, D.J. *et al.* Endoscopic transpapillary therapy for disrupted pancreatic duct and peripancreatic fluid collections. *Gastroenterology* 1991; **100**: 1362–1370.

[12] Kozarek, R.A., Jiranek, G.C. and Traverso, L.W., Endoscopic treatment of pancreatic ascites. *Am. J. Surg.* 1994; **168**: 223–226.

